# Serine 477 plays a crucial role in the interaction of the SARS-CoV-2 spike protein with the human receptor ACE2

**DOI:** 10.1038/s41598-021-83761-5

**Published:** 2021-02-22

**Authors:** Amit Singh, Georg Steinkellner, Katharina Köchl, Karl Gruber, Christian C. Gruber

**Affiliations:** 1 Institute of Molecular Bioscience, University of Graz, 8010 Graz, Austria; 2 Innophore GmbH, 8010 Graz, Austria; 3 Field of Excellence BioHealth – University of Graz, 8010 Graz, Austria; 4 Austrian Centre of Industrial Biotechnology, 8010 Graz, Austria

**Keywords:** Molecular modelling, Viral infection

## Abstract

Since the worldwide outbreak of the infectious disease COVID-19, several studies have been published to understand the structural mechanism of the novel coronavirus SARS-CoV-2. During the infection process, the SARS-CoV-2 spike (S) protein plays a crucial role in the receptor recognition and cell membrane fusion process by interacting with the human angiotensin-converting enzyme 2 (hACE2) receptor. However, new variants of these spike proteins emerge as the virus passes through the disease reservoir. This poses a major challenge for designing a potent antigen for an effective immune response against the spike protein. Through a normal mode analysis (NMA) we identified the highly flexible region in the receptor binding domain (RBD) of SARS-CoV-2, starting from residue 475 up to residue 485. Structurally, the position S477 shows the highest flexibility among them. At the same time, S477 is hitherto the most frequently exchanged amino acid residue in the RBDs of SARS-CoV-2 mutants. Therefore, using MD simulations, we have investigated the role of S477 and its two frequent mutations (S477G and S477N) at the RBD during the binding to hACE2. We found that the amino acid exchanges S477G and S477N strengthen the binding of the SARS-COV-2 spike with the hACE2 receptor.

## Introduction

**Figure 1:**
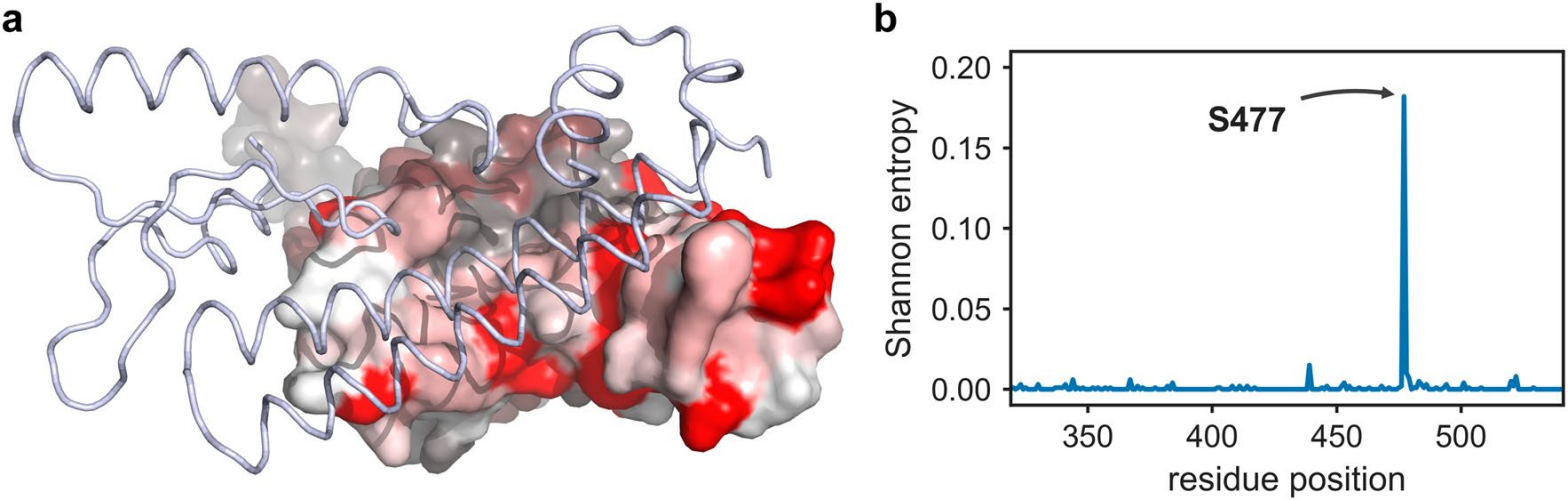
Sequence analysis for reported mutations in RBD. (**a**) Surface representation of the RBD coloured according to the conservation score at the respective residue position. Conserved residues are shown in white and shades of red represent increasing variability. A portion of hACE2 is shown in a light blue ribbon representation. (**b**) Reported mutations in RBD shown as Shannon entropy vs. residue position. The Shannon entropy is given by $H = - \mathop \sum \limits_{i = 1}^{M} P_{i} ln_{2} P_{i}$, where M is the total number of residue positions and $P_{i}$ corresponds to probability of residue at position *i*. A higher entropy value indicates a less conserved position.

In the recent global outbreak of COVID-19, more than 80 million individuals have as yet been infected with this severe acute respiratory syndrome coronavirus 2 (SARS-CoV-2). SARS-CoV-2 is responsible for around 1.8 million deaths as of December 2020. This novel virus is a member of structurally “crowned” viruses, first appreciated and defined as coronavirus in the 1960’s when Tyrrell and Bynoe discovered the first human coronaviruses (HCoVs)^1^. These single-stranded RNA viruses have round enveloped virions and are covered by trimeric aggregates of spike (S) proteins. The S protein is a type I fusion protein and is involved in receptor binding, eventually leading to virus fusion with the host cell. Specifically, the SARS-CoV-2 spike receptor binding domain (RBD) is known to interact with the human angiotensin-converting enzyme 2 (hACE2) receptor^2,3^. A conformational change in the trimeric S protein exposes one of its RBD domains in an active “up” state and triggers the binding of the receptor binding motif (RBM, residues 437 to 508) to hACE2^4,5^. Subsequently, the S protein is also recognized by the immune cells as a primary antigenic target to neutralize the virus. However, favourable mutations for the virus could be acquired through natural selection, following this as of now hundreds of new mutations were found on different residue positions of the S protein. While preparing potential targets of the SARS-CoV-2 proteome for our recently published large-scale virtual screening^6,7^ aiming to identify potential binders and inhibitors, we analysed the diversity of the SARS-CoV-2 genome landscape: Till 30th August 2020, among 73,042 reported SARS-CoV-2 spike sequences on GISAID we have found that a total of 185 amino acid substitutions are reported on the 223 residues long RBD and 68 exchanges are reported in the 72 residues long RBM (Fig. 1a)^8^. Within the SARS-CoV-2 spike protein, residue D614 is a highly variable site, whereas residue S477 has the highest number of mutation events within the RBD (Fig. 1b). Interestingly, S477N occurs very frequently alongside the D614G variant which has been reported to be associated with a very high viral load^9^, structurally these two sites are located distinctly in the spike protein, but the very high frequency of co-occurrence among these two sites needs to be investigated.

Further, the rate of mutation in the RBD is posing a greater threat since the interaction of this domain to hACE2 is the key to entering a host cell and recent studies are highlighting that several mutations in the RBD indeed strengthened the SARS-CoV-2 infectivity^10–14^. Each mutation is said to have an impact on the protein–protein interaction interface in the heterodimeric complex of hACE2 and the RBD. However, the site in the RBD, which looks very promising to investigate, is the residue S477. As of 30th August 2020, position 477 has the highest frequency of mutation in the RBD (Fig. 1b) with reported substitutions to G(2) I(7) N(3400) R(2) T(2) X(20), where X denotes any amino acid. Deep mutational scanning of the RBD has shown that a mutation at S477 has a potential to affect the stability of the RBD as well as its binding to hACE2^15^, whereas the structural effect of residue S477 in context of intra-RBD interactions is lesser understood while it is reported that the residues S477 and T478 in SARS-CoV-2 are responsible for specific and efficient interactions with hACE2 and provide an edge over SARS-CoV^16,17^. Therefore, we investigate the structural contribution of residue S477 in the RBD in order to understand its role in hACE2 binding.

Unbound proteins are predisposed to undergo conformational fluctuations which are relevant for protein–protein interactions and intra-protein interactions and have a profound role in presenting surface exposed residues in inter-protein interactions^18–20^. A structural analysis of the unbound RBD is therefore a very critical step for designing a potent inhibitor or an antibody to potentially block hACE2 and RBD interactions. In this study, we are investigating the intrinsic motion of the RBD in an unbound state using the normal mode analysis (NMA) technique. NMA is a time dependent harmonic approximation of internal motion in proteins around an energy minimum^21–23^ and proved to be a very handy tool to study the harmonic motions relating to protein dynamics and conformational fluctuations^21,22,24^. NMA has been used previously to resolve the domain motions and dynamical correlation among protein residues^24,25^.

## Results

### Flexibility analysis highlighted a highly correlated domain motion in the RBD

**Figure 2:**
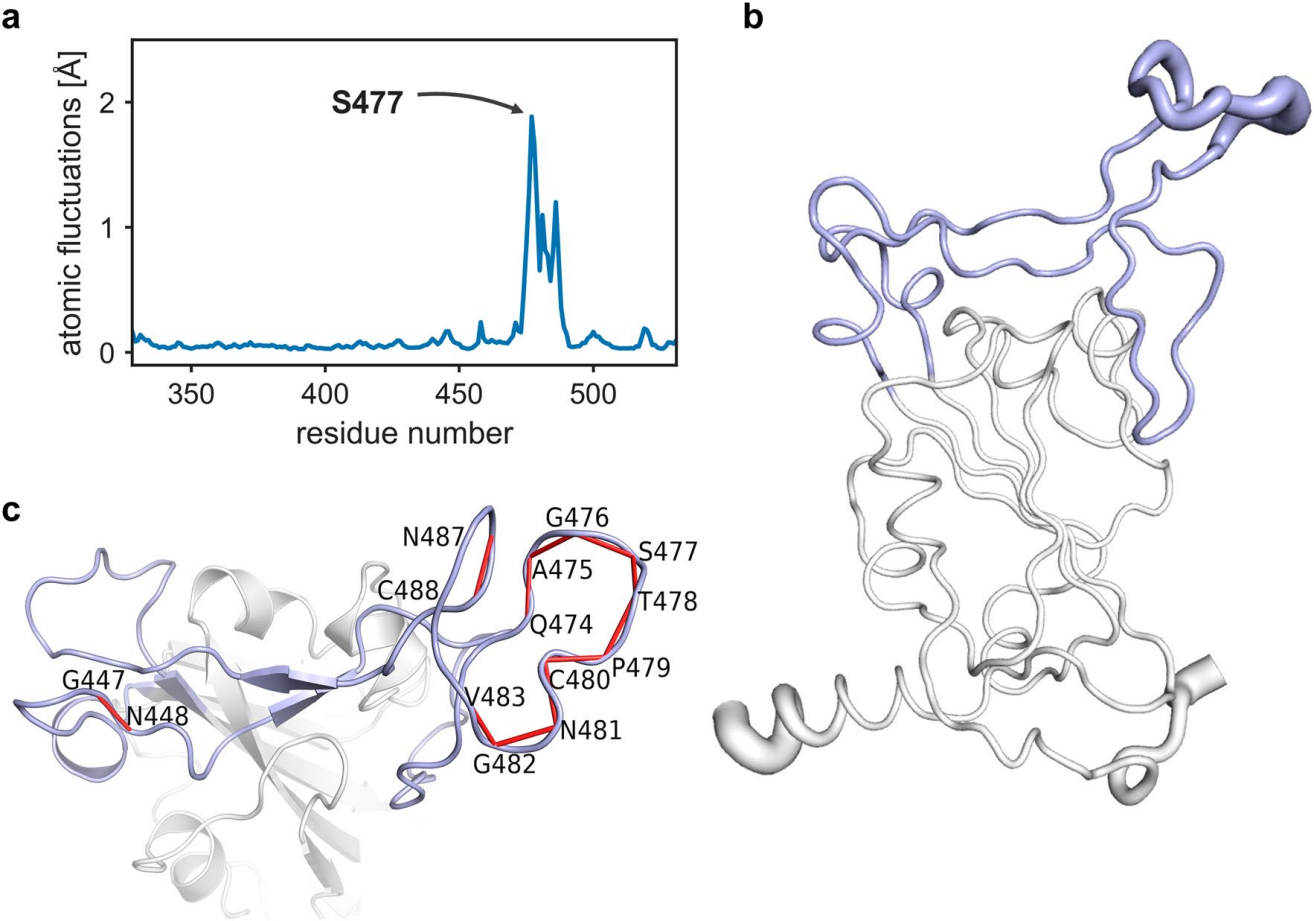
DynaMut flexibility analysis performed over the first 10 non-trivial modes of the molecule. (**a**) Residue wise atomic fluctuations in RBD. (**b**) Structure of the RBD in a ‘sausage-style’ representation with the thickness of the tube indicating the magnitude of the fluctuations. The RBM is shown in light blue. (**c**) Annotation of dynamical cross correlations among residues of the RBD, with the RBM again shown in light blue. Only the highly correlated pairs of residues with a correlation coefficient of (≥ 0.9) are annotated and connected by red lines.

The RBD is very efficient in finding its attachment surface on hACE2 and is therefore SARS-CoV-2’s first line tool to explore its potential binding target on a human cell. It has been shown that the flexibility of surface exposed residues plays a key role in the recognition of potential binding sites during protein–protein interactions^26–28^. Therefore, we performed a detailed flexibility analysis to identify “flexible” and “rigid” regions of the SARS-CoV-2 RBD. We found significant differences among the dynamics of those residues, especially in the receptor binding motif (RBM, residues 437 to 508) of the RBD (Fig. 2). The key flexible region within the RBM covers the residues from 475 to 487, which might have a key role for an induced fit binding with hACE2. It has also been reported that the region, which corresponds to residues 475–483 in RBD, is a site of interest due to a higher frequency of mutations in this region^9,15^.

Normal mode flexibility analyses have shown an array of highly dynamic residues from residue 475 to 487 in the RBM. Surprisingly, these residues have a very high dynamical pair correlation among them with a correlation coefficient of ≥ 0.9 (Fig. 2c). Apart from RBM, RBD has no other highly correlated dynamical pair of residues in it.

Specifically, we have noticed an interesting site among these highly flexible residues in the RBM, the residue S477, preceded by G476. A mutation corresponding to S477G results in two consecutive Gly residues at position 476 and 477, with the resulting arrangement A475-G476-S477G (AGG), This AGG motif might favour easier structural arrangements of side chains which are otherwise restricted^29,30^. Our detailed NMA suggested that there is a flexible domain of highly correlated dynamical residues on RBM, and local amino acid exchanges S477G and S477N have a very high potential to affect local conformation significantly upon binding. Further using MD simulations, we are investigating a critical loop on RBM, which has a prominent role in binding with hACE2.

### Volumetric map reveals interaction hotspot on hACE2

**Figure 3:**
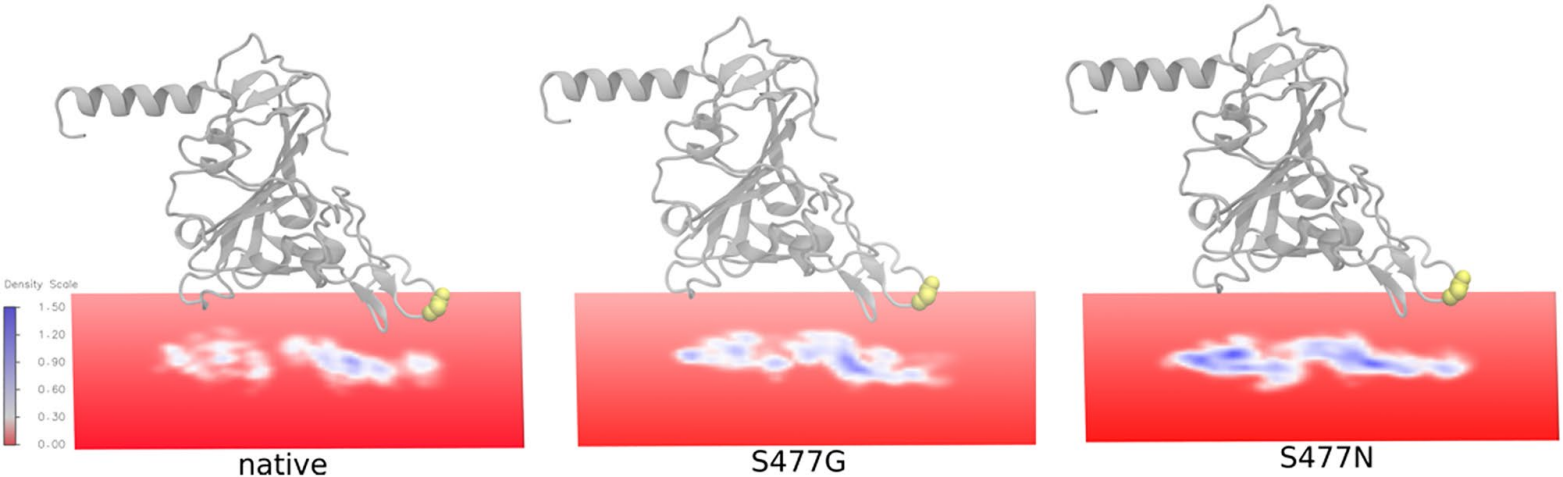
Volumetric map analysis of 100 ns trajectory showing the hACE2 residues within 5 Å of RBD. Native RBD and the variants S477G and S477N are shown as grey cartoon representations with residue 477 highlighted in yellow. The Volumetric map was created by using the VMD^53^ Volmap toolkit that generated a map of the weighted atomic density of every ACE2 atom within 5 Å of RBD at each gridpoint. This is done by replacing each atom in the selection with a normalized gaussian distribution of width (standard deviation) equal to its atomic radius.

To verify the impact of the mutation S477G in affecting the binding with hACE2, molecular dynamics simulations (MD) have been carried out to obtain volumetric maps to visualize the captured signature of inter atomic contacts. We performed 100 ns MDs with the native hACE2:RBD complex and the hACE2:RBD S477G and S477N variant complexes. Based on the atomic coordinates from these 100 ns trajectories a volumetric map was created by selecting the hACE2 residues within 5 Å of the RBD. The resulting maps, as shown in Fig. 3, reveal notable differences: There is a pronounced interaction hotspot on hACE2 opposite to residue S477 of the RBD in the native system as well as in the S477N variant, while it is missing in the S477G variant. This difference is most likely due to steric reasons because of the lack of side chain functional groups in glycine. Qualitatively this analysis indicated a larger “footprint” of the variants compared to native RBD (Fig. 3). In line with that, the average total numbers of interface hydrogen bonds per trajectory frame of the 100 ns simulations are 5.7, 6.0, 6.7, for the native, S477G and S477N variants, respectively. The binding interface of 100 ns equilibrated RBD:hACE2 complex annotated with the residues involved in inter-chain interactions (Fig. S1) also shows the divergent interactions at the interface. Therefore, further analyses were carried out to understand the structural impact of the amino acid exchange at position 477.

### S477 variants are showing contrasting change in flexibility upon binding to hACE2

**Figure 4:**
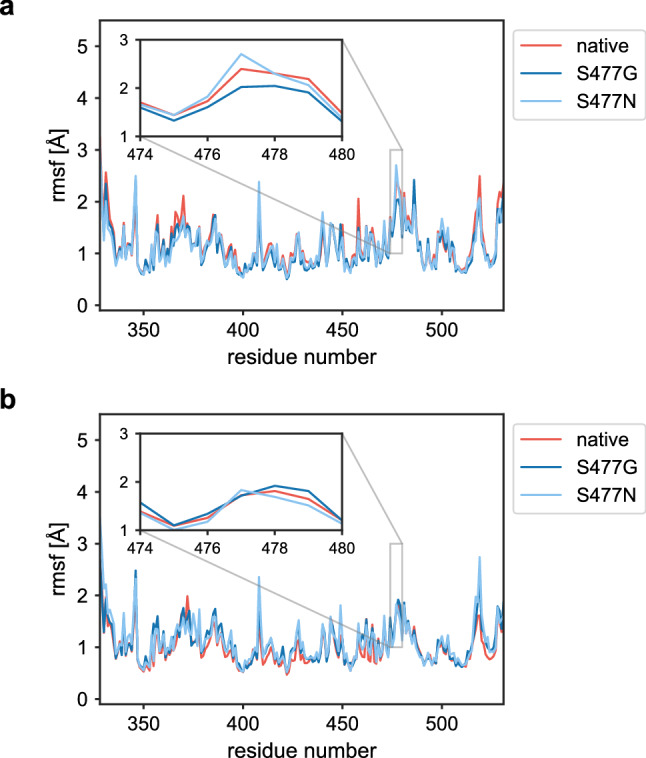
Comparison of residue wise root mean square fluctuations. (**a**) Unbound RDB, (**b**) in complex with hACE2. Each graph is averaged over three independent 10 ns simulations, the standard error is shown as semi-transparent bands. For clarity, values for the 10 N- and C-terminal residues of RBD are not shown.

In MD analyses, the root mean square fluctuation (RMSF) is a broadly used tool to identify residue-specific-flexibility^31,32^. Plotting the RMSF of each residue in the RBD, in its unbound form as well as in complex with hACE2, we noticed an overall decrease in flexibility of the RBD upon binding with hACE2 compared to the unbound RBD (Fig. 4). This decrease was particularly pronounced in the region around residue 477, suggesting an active participation of this residue at the interaction interface. Although we expected an increased local flexibility of the loop in the S477G variant, this hypothesis surprisingly turned out not to be true.

### S477G delayed the detachment of RBD from hACE2

Steered molecular dynamics (SMD) is an advanced version of MD simulations, widely used to study the binding and unbinding events among biomolecules in structural biology^33–36^, where the dissociation process is being accelerated using an external forced pulling via a harmonic force constant. SMD simulations have proved their worthiness in resolving the effect of single point mutation on protein–protein interaction interfaces^37,38^.

**Figure 5:**
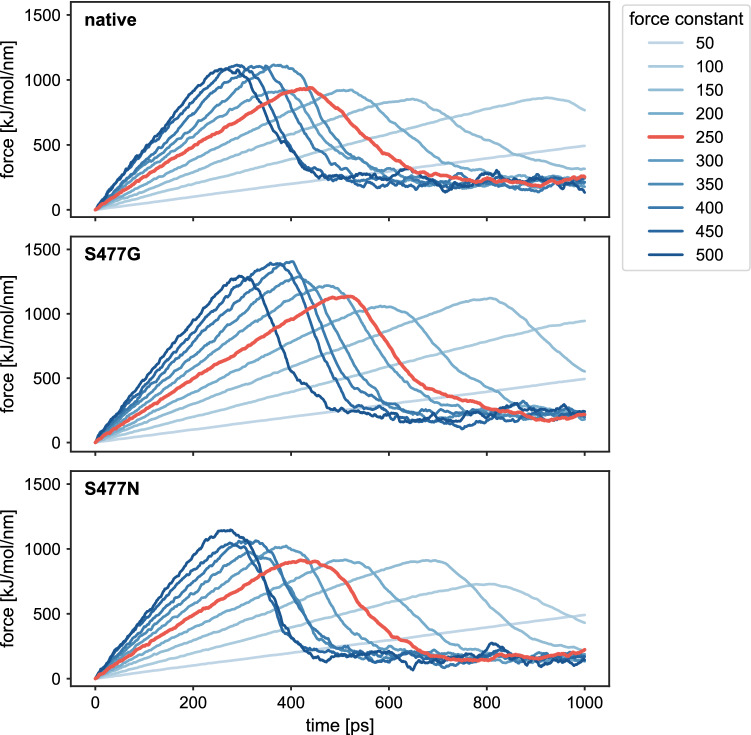
Influence of the spring constant on the steering force of RBD. The steering force along the SMD trajectories is shown for different spring force constants, given in kJ/mol/nm^2^. Increasing force constants are indicated by increasing depths of blue. The curves resulting from SMD simulations with a force constant of 250 kJ/mol/nm^2^, which were later used for umbrella sampling, are highlighted in red.

To study the impact of the amino acid exchanges at S477 in the detachment and association process of the spike protein to hACE2, an SMD simulation was carried out. The forced dissociation of the RBD from hACE2 depends on the spring constant and tension exerted by the surroundings (Fig. 5). Interestingly, there is a significant difference in the rupture force between the RBD and its variants S477G and S477N throughout different force constants. The chosen spring constant of 250 kJ/mol/nm^2^ allows for a consistent and smooth transition between bound and unbound complex. A final center-of-mass (COM) distance between hACE2 and the RBD of approximately 14 nm was achieved.

**Figure 6:**
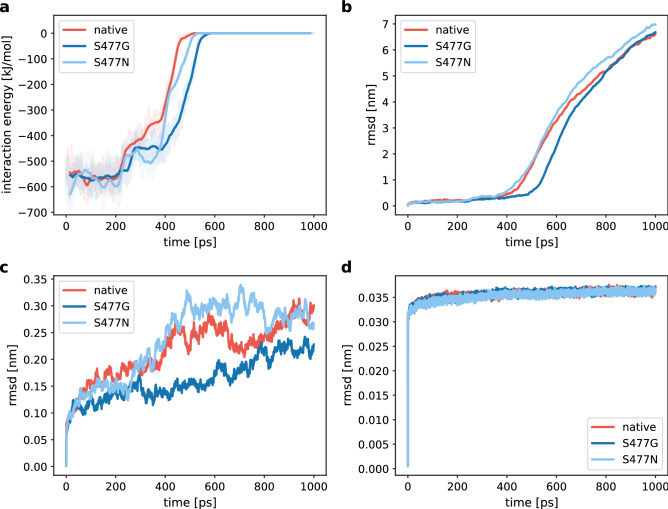
Energy and structural changes during SMD simulations with a spring force constant of 250 kJ/mol/nm^2^. (**a**) Time dependence of the interaction energy between hACE2 and RBD. The shaded areas represent non-averaged data (frame rate 0.1 ps), whereas the lines represent window averages of 300 frames. (**b**) Comparison of the rmsd of the backbone carbon atoms in the RBD:hACE2 complex relative to the initial frame. (**c**) Comparison of the rmsd of the carbon atoms in the RBD backbone relative to the initial frame. (**d**) Comparison of the rmsd of the carbon atoms in the hACE2 backbone relative to the initial frame.

**Figure 7:**
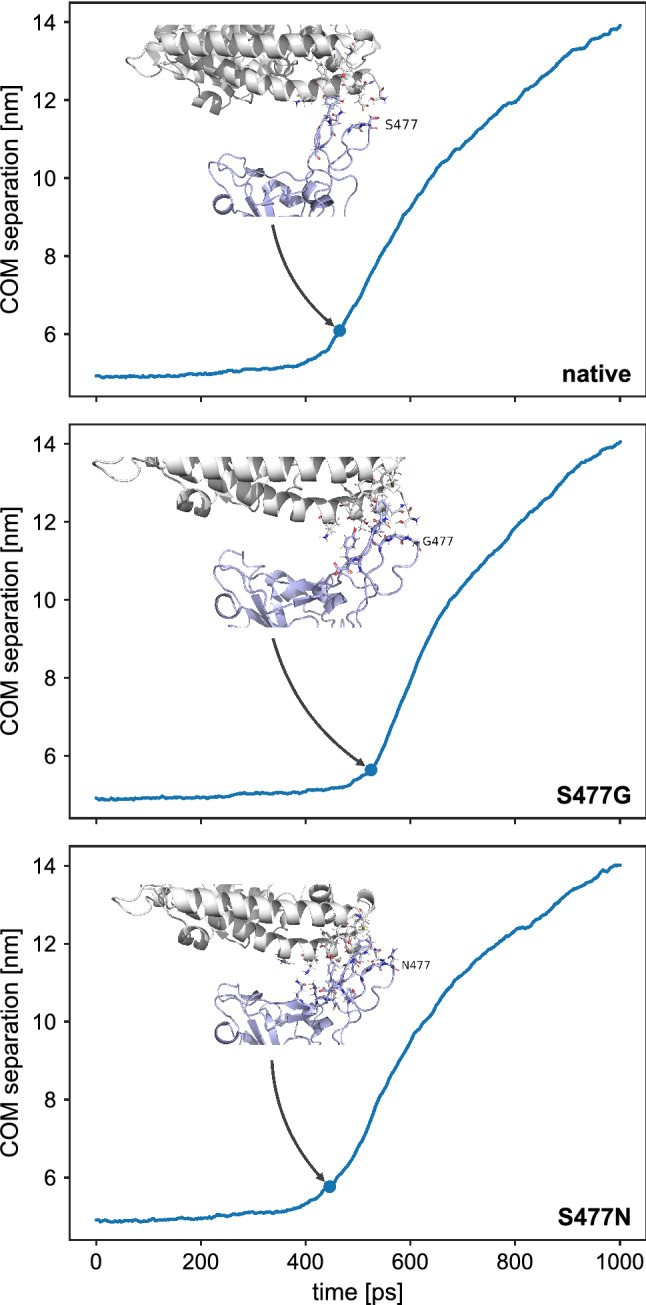
Snapshots of the hACE2:RBD interaction interface at the force rupturing event during SMD simulation. The structure of hACE2 is shown in grey, whereas the RBD is shown in light blue. Interacting residues on both binding partners are shown in a ball-and-stick representation. Structures and values for the COM separation were extracted from an SMD simulation with a spring force constant of 250 kJ/mol/nm^2^. The breakage point was defined as a COM separation of 1 nm larger than the separation at the start of the simulation.

**Figure 8:**
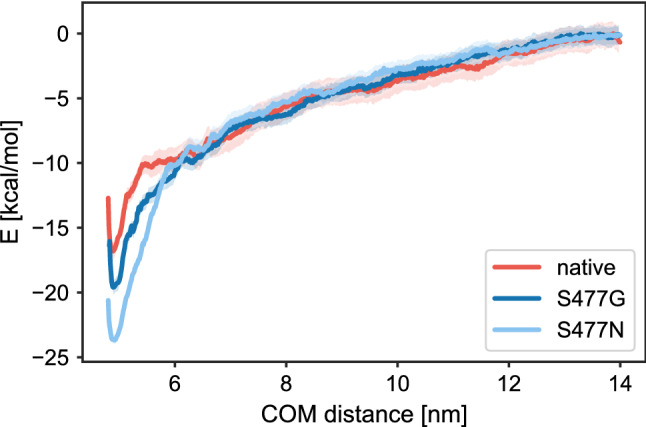
Potential of mean force calculation from SMD simulation of RBD and its S477 variants. The standard deviation is indicated by a semi-transparent band in the same colour.

Energy and conformational changes show that the rupture force varied greatly between the RBD and its S477 variants. Van der Waals energies show lesser fluctuations in the event of forceful dissociation of RBD from hACE2, whereas in the beginning the electrostatic forces dominated (Fig. S2). The complex of native RBD and hACE2 started dissociating after 400 ps (Fig. 6), S477N and hACE2 started dissociating at 390 ps, whereas the S477G variant delayed the disassociation process with hACE2 for around 100 ps. To further confirm the time point of disassociation of the RBD:hACE2 complex, we plotted the distance between RBD and hACE2 *vs.* time, indicating that the S477G RBD variant is significantly delaying the disengagement process (Fig. 7). Potential of mean force calculations (Fig. 8) show that the S477N variant features the highest binding affinity with hACE2 compared to its native RBD and S477G (Table 1). Our results are in line with experimentally reported changes in the binding affinity of S477G and S477N variants^15^.

**Table 1:** Binding free energies for native and S477 variants with standard deviation.

System	ΔG_bind_ (kcal/mol)	ΔΔG_bind_ (kcal/mol)
Native	− 16.8 ± 1.4	–
S477G	− 19.6 ± 1.1	− 2.8 ± 1.8
S477N	− 23.7 ± 0.9	− 6.9 ± 1.6

To visualize the changes in the dissociation process we took structural snapshots at the breaking point for both, the RBD and its S477 variants. The inset structural annotation as shown in Fig. 8 at the breaking point indicates that the interaction with the highly flexible loop residues of the RBD will remain until the end of the unbinding event.

## Discussion

Our flexibility and correlational analyses reveal a highly dynamic loop in the SARS-CoV-2 spike protein RBM from residue 475 to 485. It is predicted by induced fit theory^39^ that the binding event induces a conformational change at the interaction site, and biased dynamics of these highly correlated residues^14^ at the RBM are emphasizing their relevance for hACE2 binding. In fact, loops are the most common structural features found at protein–protein interaction interfaces^40^. Forced pulling dynamics revealed that this highly flexible loop remained interacting with hACE2 till the end of the rupturing event, suggesting that it provides the required mobility to mediate the hACE2:RBD interaction. Following this, it is worth considering that any alteration in the flexibility of this loop could have a strong impact on the binding of the RBD with hACE2. The comparative analysis of the RBD and its S477 variants highlights the potential effects of single amino acid substitutions on protein–protein interactions. Both single mutations of S477 have shown an increased binding affinity for hACE2. Based on our flexibility and SMD analyses, we are emphasizing that any mutation subject to a structural change in this region could potentially be critical for the interaction between RBD and hACE2.

## Methodology

### Template crystal structure

As a starting point the recently published crystal structure of the SARS-CoV-2 spike receptor-binding domain bound to the hACE2 receptor obtained from PDB (PDB-Code:6M0J) having a resolution of 2.47 Å^41^ was used. Missing heavy atoms, residues and hydrogen were then modelled by using the web based CHARMM package^42,43^. The amino acid exchanges S477N and S477G were modelled using the standalone CHARMM package.

### Simulation system details

#### System 1—unbound RBD

The coordinates of the RBD and its S477 variants were extracted from the above mentioned fully prepared structure. All structures were subjected to an energy minimization step followed by 100 ps of NVT. A 100 ns equilibration run was performed to relax the interface side-chain conformations constrained by the hACE2. This relaxed unbound RBD structure was used for the NMA on the DynaMut server^44^.

For a comparative flexibility analysis based on the root-mean-square fluctuation (RMSF), all three structures were further subjected to three independent 10 ns production runs each.

#### System 2—complex of hACE2:RBD

The coordinates of the hACE2:RBD complexes (native and S477 variants) were taken from the above-mentioned template crystal structure. All complexes were further subjected to an energy minimization step followed by 100 ps of NVT and a 100 ns equilibration run. These equilibrated structures were used as input for the SMD simulations.

As above, the thus equilibrated complex structures were further subjected to three independent 10 ns production runs each for RMSF analyses.

### Molecular dynamics simulation details

Each system was placed in the center of a box with a minimum of 10 Å distance from the edge of the box, to avoid imaginary interactions, followed by solvation and system neutralization by adding ions. Potential energy terms for the protein and ions were derived from CHARMM36^45^ all atom additive force field, and TIP3P^46^ potential was used to represent water molecules.

All classical simulations were performed using the Gromacs 2018^47,48^ package with enhanced GPU support. A step size of 2 fs was chosen with a 10 Å cutoff for non-bonded interactions and Particle Mesh Ewald (PME)^49^ summation was used to deal with long range interactions. All covalent bonds involving hydrogen were constrained using LINCS^50^. The temperature was maintained at 300 K with a Velocity-rescaling algorithm^51^ using two indexed groups: protein and non-protein, for effective temperature coupling. Pressure coupling was applied isotropically at 1 bar with a weak coupling constant of 2 ps and an isothermal compressibility value of 4.5 × 10^–5^ bar^−1^.

### Steered MD simulation details

For SMD, the structures were placed in a rectangular box with the dimensions (100 Å × 100 Å × 350 Å)—sufficient to avoid imaginary interactions and providing space for pulling simulations to take place along the Z-axis—followed by solvation and neutralization by adding ions. Subsequently, bad contacts were removed by energy minimization, followed by a 100 ps NVT and a 500 ps NPT equilibration. After equilibration, position restraints were removed from the RBD whereas hACE2 remained as an immobile reference for the pulling simulations with position restraint of 1000 kJ/mol/nm^2^ on heavy atoms in xyz direction. In each of the 10 different pulling simulations, the RBD was pulled away from the hACE2 along the Z-axis over 1000 ps with a fixed spring constant value, starting from 50 kJ/mol/nm^2^ for the first simulation to 500 kJ/mol/nm^2^ for the 10th simulation, with an increment of 50 kJ/mol/nm^2^ in each step between the first and last simulations. A pull rate of 0.1 Å/ps has been used throughout all 10 pulling simulations. All pulling simulations were performed with the GROMACS package.

To generate the starting configurations for umbrella sampling we have chosen trajectories resulting from the pulling simulations with the spring constant of 250 kJ/mol/nm^2^. Symmetrical distribution of sampling windows was used with a window spacing of 0.1 nm, up to a 14 nm center-of-mass (COM) separation. Such finer spacing resulted in 90 windows at different COM distances. In each window, 5 ns of MD was performed for a total simulation time of 450 ns utilized for umbrella sampling. Finally, analysis of the umbrella sampling was performed with the weighted histogram method (WHAM)^52^ with 300 bootstrapping steps in order to estimate standard deviations of the binding free energy.

### Normal mode analysis (NMA)

Normal modes are represented by eigenvectors of the Hessian matrix and eigenvalues are the squares of the associated frequencies. Each eigenvector describes a state of the protein where all Cα atoms are oscillating with the same characteristic frequency.

Using the characteristic frequency of each mode, atomic fluctuations have been calculated, given by:

$$\left\langle {\Delta x_{i}^{2} } \right\rangle = \frac{{k_{B} T}}{m} + \mathop \sum \limits_{j = 1}^{3N - 6} \frac{{a_{ij}^{2} }}{{\omega_{j}^{2} }},$$

where $\left\langle {\Delta x_{i}^{2} } \right\rangle$ is the time averaged square displacement of atom i, $\omega_{j}$ is the frequency of mode j, $a_{ij}$ is the displacement of atom i under mode j, and N is the number of residues.

The DynaMut online server takes advantage of the graph-theory and normal mode analysis (NMA) and has been employed to analyse and visualize the RBD dynamics, to generate a consensus prediction of protein motion and flexibility^44^.

### Root mean square fluctuations (RMSF)

RMSF analysis described the average deviation of a residue over a time from a reference position. Using RMSF the portions of the structure that are flexible or rigid could be identified. After removing the periodic boundary conditions using *Gromacs trjconv* tool, the gmx rmsf module has been used to calculate RMSF using the following equation:

$$RMSF_{i} = \sqrt {\frac{1}{T}\mathop \sum \limits_{{t_{j = 1} }}^{T} \left| {r_{i} (t_{j} ) - r_{i}^{ref} } \right|^{2} ,}$$

where T is the total time of trajectory, $t_{j}$ is the time at jth frame of trajectory, $r_{i}$ is the position of the atom i and $r_{i}^{ref}$ is the initial reference position of the atom i.

### Root mean square deviations (RMSD)

The RMSD of a protein is taken as the root of the average square displacement over all N Cα atoms, very often the measure of how much the protein conformation has changed. After removing the periodic boundary conditions using the *Gromacs trjconv* tool, the gmx rms module has been used to calculate the RMSD using the following equation:

$$RMSD\left( t \right) = \sqrt {\frac{1}{M}\mathop \sum \limits_{i = 1}^{N} m_{i} \left| {r_{i} \left( t \right) - r_{i}^{ref} } \right|^{2} ,}$$

where M is the total mass of the protein, N is the total number of amino acids, $m_{i}$ is the mass of amino acid *i,* $r_{i} \left( t \right)$ is the position of the atom i at time t and $r_{i}^{ref}$ is the initial reference position of the atom i.

## Supplementary Information

Supplementary Information.

Supplementary Video 1.

## Data Availability

The molecular dynamics trajectories, input files and parameter files generated within this work are available for download at https://doi.org/10.6084/m9.figshare.13234529.v1.

## References

[1] Tyrrell D, Bynoe M (1965). Cultivation of a novel type of common-cold virus in organ cultures. BMJ.

[2] Shang J (2020). Structural basis of receptor recognition by SARS-CoV-2. Nature.

[3] Shang J (2020). Cell entry mechanisms of SARS-CoV-2. Proc. Natl. Acad. Sci..

[4] Wrapp D (2020). Cryo-EM structure of the 2019-nCoV spike in the prefusion conformation. Science.

[5] Walls AC (2020). Structure, function, and antigenicity of the SARS-CoV-2 spike glycoprotein. Cell.

[6] Gruber, C. C. & Steinkellner, G. Wuhan coronavirus 2019-nCoV—What we can find out on a structural bioinformatics level. 24044224 Bytes (2020). 10.6084/M9.FIGSHARE.11752749.V3.

[7] Gorgulla C (2020). A multi-pronged approach targeting SARS-CoV-2 proteins using ultra-large virtual screening. ChemRxiv..

[8] Shu Y, McCauley J (2017). GISAID: Global initiative on sharing all influenza data—From vision to reality. Eurosurveillance.

[9] Korber B (2020). Tracking changes in SARS-CoV-2 spike: Evidence that D614G increases infectivity of the covid-19 virus. Cell.

[10] Chen, J., Wang, R., Wang, M. & Wei, G.-W. Mutations strengthened SARS-CoV-2 infectivity. Preprint at http://arXiv.org/2005.14669 (2020). 10.1016/j.jmb.2020.07.009 PMC7375973 32710986

[11] Ozono S (2020). Naturally mutated spike proteins of SARS-CoV-2 variants show differential levels of cell entry. BioRxiv..

[12] Ou J (2020). Emergence of RBD mutations in circulating SARS-CoV-2 strains enhancing the structural stability and human ACE2 receptor affinity of the spike protein. BioRxiv..

[13] Wang Y, Liu M, Gao J (2020). Enhanced receptor binding of SARS-CoV-2 through networks of hydrogen-bonding and hydrophobic interactions. Proc. Natl. Acad. Sci. U.S.A..

[14] Spinello A, Saltalamacchia A, Magistrato A (2020). Is the rigidity of SARS-CoV-2 spike receptor-binding motif the hallmark for its enhanced infectivity? Insights from all-atom simulations. J. Phys. Chem. Lett..

[15] Starr TN (2020). Deep mutational scanning of SARS-CoV-2 receptor binding domain reveals constraints on folding and ACE2 binding. Cell.

[16] Wang Q (2020). Receptor utilization of angiotensin-converting enzyme 2 (ACE2) indicates a narrower host range of SARS-CoV-2 than that of SARS-CoV. Transbound Emerg. Dis..

[17] Ghorbani M, Brooks BR, Klauda JB (2020). Critical sequence hotspots for binding of novel coronavirus to angiotensin converter enzyme as evaluated by molecular simulations. J. Phys. Chem. B.

[18] Li X, Keskin O, Ma B, Nussinov R, Liang J (2004). Protein–protein interactions: hot spots and structurally conserved residues often locate in complemented pockets that pre-organized in the unbound states: Implications for docking. J. Mol. Biol..

[19] Tobi D, Bahar I (2005). Structural changes involved in protein binding correlate with intrinsic motions of proteins in the unbound state. Proc. Natl. Acad. Sci..

[20] Jayashree S, Murugavel P, Sowdhamini R, Srinivasan N (2019). Interface residues of transient protein-protein complexes have extensive intra-protein interactions apart from inter-protein interactions. Biol. Direct.

[21] Levitt M, Sander C, Stern PS (1985). Protein normal-mode dynamics: Trypsin inhibitor, crambin, ribonuclease and lysozyme. J. Mol. Biol..

[22] Ben-Avraham D (1993). Vibrational normal-mode spectrum of globular proteins. Phys. Rev. B.

[23] Tasumi M, Takeuchi H, Ataka S, Dwivedi A, Krimm S (1982). Normal vibrations of proteins: Glucagon. Biopolym. Original Res. Biomol..

[24] Case DA (1994). Normal mode analysis of protein dynamics. Curr. Opin. Struct. Biol..

[25] Hinsen K (1998). Analysis of domain motions by approximate normal mode calculations. Proteins Struct. Funct. Bioinform..

[26] Rajamani D, Thiel S, Vajda S, Camacho CJ (2004). Anchor residues in protein–protein interactions. Proc. Natl. Acad. Sci..

[27] Smith GR, Sternberg MJ, Bates PA (2005). The relationship between the flexibility of proteins and their conformational states on forming protein–protein complexes with an application to protein–protein docking. J. Mol. Biol..

[28] Jubb H, Blundell TL, Ascher DB (2015). Flexibility and small pockets at protein–protein interfaces: new insights into druggability. Prog. Biophys. Mol. Biol..

[29] Vila R, Ponte I, Jiménez MA, Rico M, Suau P (2002). An inducible helix–Gly–Gly–helix motif in the N-terminal domain of histone H1e: A CD and NMR study. Protein Sci..

[30] Ho BK, Brasseur R (2005). The Ramachandran plots of glycine and pre-proline. BMC Struct. Biol..

[31] Shaw DE (2010). Atomic-level characterization of the structural dynamics of proteins. Science.

[32] Maiorov VN, Crippen GM (1994). Significance of root-mean-square deviation in comparing three-dimensional structures of globular proteins. J. Mol. Biol..

[33] Izrailev S, Deuflhard P, Hermans J, Leimkuhler B, Mark AE, Reich S, Skeel RD (1999). Steered molecular dynamics. Computational Molecular Dynamics: Challenges, Methods, Idea.

[34] Isralewitz B, Gao M, Schulten K (2001). Steered molecular dynamics and mechanical functions of proteins. Curr. Opin. Struct. Biol..

[35] Cuendet MA, Michielin O (2008). Protein-protein interaction investigated by steered molecular dynamics: the TCR-pMHC complex. Biophys. J..

[36] Rodriguez RA, Yu L, Chen LY (2015). Computing protein–protein association affinity with hybrid steered molecular dynamics. J. Chem. Theory Comput..

[37] Xiao B-L (2019). Steered molecular dynamic simulations of conformational lock of Cu, Zn-superoxide dismutase. Sci. Rep..

[38] Mino G, Baez M, Gutierrez G (2013). Effect of mutation at the interface of Trp-repressor dimeric protein: A steered molecular dynamics simulation. Eur. Biophys. J..

[39] Koshland DE (1995). The key-lock theory and the induced fit theory. Angew. Chem. Int. Ed. Engl..

[40] Gavenonis J, Sheneman BA, Siegert TR, Eshelman MR, Kritzer JA (2014). Comprehensive analysis of loops at protein-protein interfaces for macrocycle design. Nat. Chem. Biol..

[41] Lan J (2020). Structure of the SARS-CoV-2 spike receptor-binding domain bound to the ACE2 receptor. Nature.

[42] Jo S, Kim T, Iyer VG, Im W (2008). CHARMM-GUI: A web-based graphical user interface for CHARMM. J. Comput. Chem..

[43] Brooks BR (2009). CHARMM: The biomolecular simulation program. J. Comput. Chem..

[44] Rodrigues CH, Pires DE, Ascher DB (2018). DynaMut: Predicting the impact of mutations on protein conformation, flexibility and stability. Nucleic Acids Res..

[45] Huang J, MacKerell AD (2013). CHARMM36 all-atom additive protein force field: Validation based on comparison to NMR data. J. Comput. Chem..

[46] Jorgensen WL, Chandrasekhar J, Madura JD, Impey RW, Klein ML (1983). Comparison of simple potential functions for simulating liquid water. J. Chem. Phys..

[47] Abraham, M., Spoel, D., Lindahl, E., Hess, B. & Team, T. *GROMACS User Manual Version 2018* (2018).

[48] Van Der Spoel D (2005). GROMACS: Fast, flexible, and free. J. Comput. Chem..

[49] Darden T, York D, Pedersen L (1993). Particle mesh Ewald: An N.log (N) method for Ewald sums in large systems. [0J. Chem. Phys..

[50] Hess B, Bekker H, Berendsen HJ, Fraaije JG (1997). LINCS: A linear constraint solver for molecular simulations. J. Comput. Chem..

[51] Bussi G, Donadio D, Parrinello M (2007). Canonical sampling through velocity rescaling. J. Chem. Phys..

[52] Li Z, Fichthorn KA, Milner ST (2016). Surfactant binding to polymer–water interfaces in atomistic simulations. Langmuir.

[53] Humphrey W, Dalke A, Schulten K (1996). VMD: Visual molecular dynamics. J. Mol. Graph..

