# Supplementary material for: Serine 477 plays a crucial role in the interaction of the SARS-CoV-2 spike protein with the human receptor ACE2
Source: Sci Rep. 2021 Feb 22;11:4320. doi: 10.1038/s41598-021-83761-5 (PMC7900180; doi:10.1038/s41598-021-83761-5)
Supplement: Supplementary file 1 — Supplementary Information. [file 41598_2021_83761_MOESM1_ESM.pdf]

Supporting information for:

Serine 477 plays a crucial role in the interaction of the SARS-CoV2 spike protein with the human receptor ACE2.

Amit Singh[1], Georg Steinkellner[1,2], Katharina Köchl[2], Karl Gruber[1,3,4*], Christian C. Gruber[1,2*]

[1]Institute of Molecular Bioscience, University of Graz, 8010 Graz, Austria

[2]Innophore GmbH, 8010 Graz, Austria

[3]Field of Excellence BioHealth – University of Graz, 8010 Graz, Austria

[4]Austrian Centre of Industrial Biotechnology, 8010 Graz, Austria

*Correspondence should be addressed to (email: karl.gruber@uni-graz.at, christian.gruber@innophore.com)

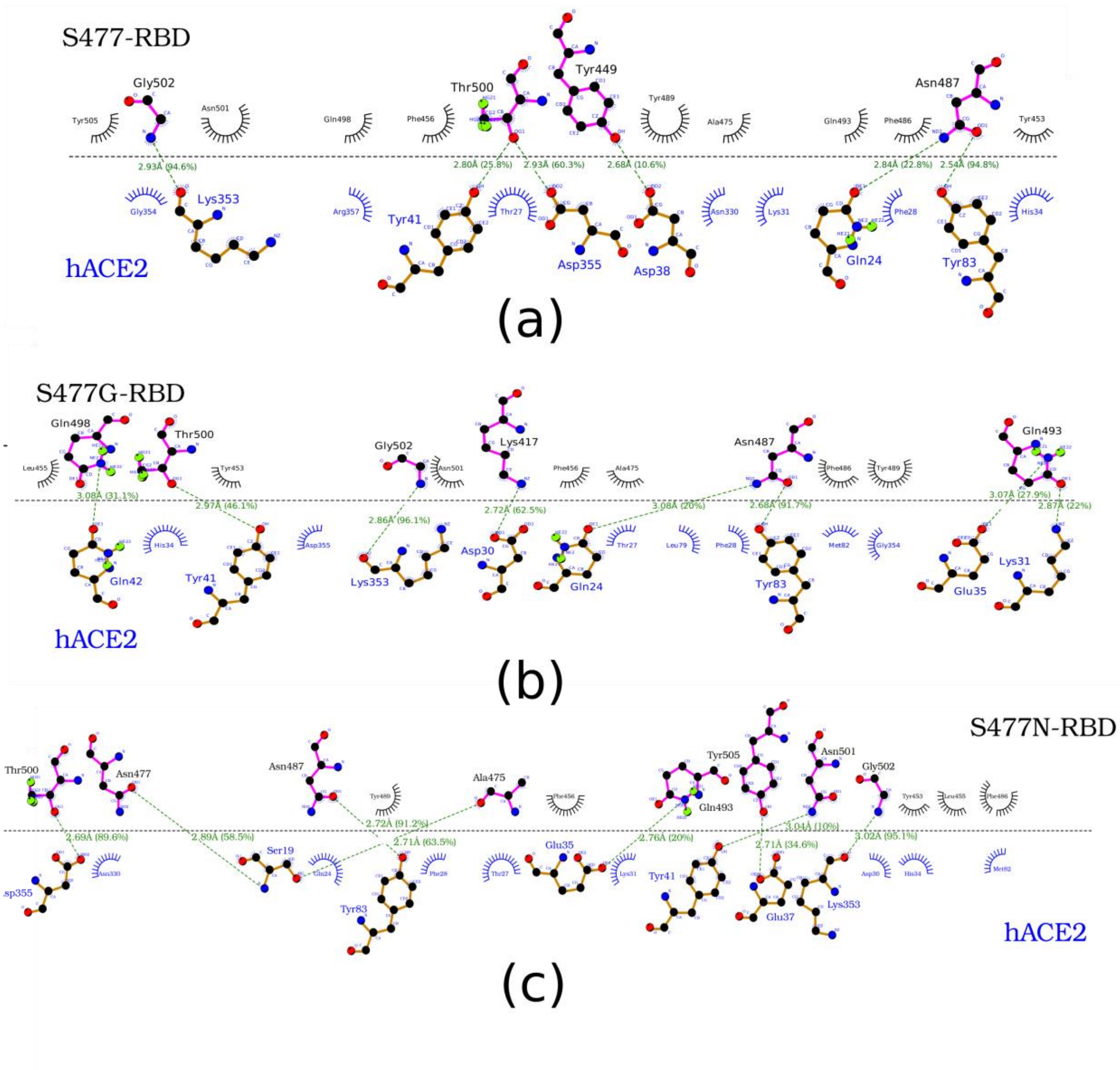

Figure S1: A closer view of the binding interface after the 100ns production run, where hydrogen bonds are shown in green dotted lines and labelled with observed distance (in Å) between donor and acceptor atoms while percentage occupancy (Frequency of occurring a hydrogen bond * 100/ Total Frames) shown in parenthesis. Residues involved in non-bonded interactions marked as bent comb drawings. Residues of hACE2 are shown in blue, whereas residues of RBD variants are shown in black. A) Native:hACE2 B) S477G:hACE2 c) S477N:hACE2. Figure plotted using Dimplot module of LigPlot+ [1].

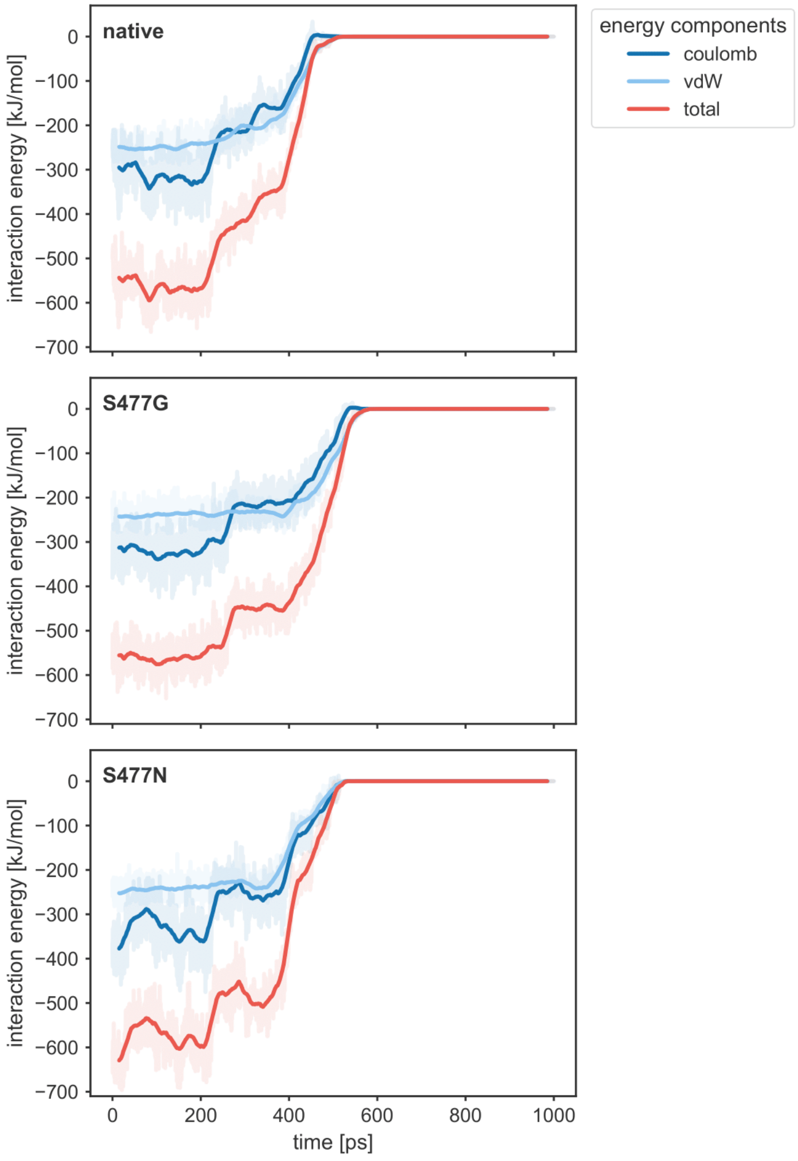

Figure S2: Energy changes during SMD simulations with a spring force constant of 250 kJ/mol/nm^2. a) Time dependence of the interaction energy between hACE2 and RBD. The shaded areas represent non-averaged data (frame rate 0.1 ps), whereas the lines represent window averages of 300 frames.

Supplementary movie 1: A movie showing the centre of mass (COM) separation between the hACE2 and RBD using a steered MD simulation with a spring force constant of 250 kJ/mol/nm^2. hACE2 is shown in a grey cartoon representation and is fixed, while RBD shown in a blue cartoon representation is pulled away. The flexible loop residues [475:485] are shown in orange.

References:

[1] Laskowski, Roman A., and Mark B. Swindells. "LigPlot+: multiple ligand–protein interaction diagrams for drug discovery." (2011): 2778-2786.
